# Lissencephaly in Shih Tzu dogs

**DOI:** 10.1186/s13028-020-00528-0

**Published:** 2020-06-20

**Authors:** Diego Noé Rodríguez-Sánchez, Giovana Boff Araujo Pinto, Edval Fernando Thomé, Vânia Maria de Vasconcelos Machado, Rogério Martins Amorim

**Affiliations:** 1 Department of Veterinary Clinics, School of Veterinary Medicine and Animal Science, São Paulo State University (UNESP), Botucatu, SP 18618-681 Brazil; 2 Department of Animal Reproduction and Veterinary Radiology, School of Veterinary Medicine and Animal Science, São Paulo State University (UNESP), Botucatu, SP 18618-681 Brazil

**Keywords:** Agyria, Arachnoid cysts, Malformation of cortical development, Pachygyria, Seizures

## Abstract

**Background:**

Lissencephaly is a brain malformation characterized by smooth and thickened cerebral surface, which may result in structural epilepsy. Lissencephaly is not common in veterinary medicine. Here, we characterize the first cases of lissencephaly in four Shih Tzu dogs, including clinical presentations and findings of magnetic resonance imaging of lissencephaly and several concomitant brain malformations.

**Case presentation:**

Early-onset acute signs of forebrain abnormalities were observed in all dogs, which were mainly cluster seizures and behavioral alterations. Based on neurological examination, the findings were consistent with symmetrical and bilateral forebrain lesions. Metabolic disorders and inflammatory diseases were excluded. Magnetic resonance imaging for three dogs showed diffuse neocortical agyria and thickened gray matter while one dog had mixed agyria and pachygyria. Other features, such as internal hydrocephalus, supracollicular fluid accumulation, and corpus callosum hypoplasia, were detected concomitantly. Antiepileptic drugs effectively controlled cluster seizures, however, sporadic isolated seizures and signs of forebrain abnormalities, such as behavioral alterations, central blindness, and strabismus persisted.

**Conclusions:**

Lissencephaly should be considered an important differential diagnosis in Shih Tzu dogs presenting with early-onset signs of forebrain abnormalities, including cluster seizures and behavioral alterations. Magnetic resonance imaging was appropriate for *ante*-*mortem* diagnosis of lissencephaly and associated cerebral anomalies.

## Background

Lissencephaly in mammals occurs due to the failure of neuroblasts to migrate to the cerebral cortex, during development [1, 2]. It is characterized by smooth cortical appearance and by the absence of surface folds (agyria) or abnormally broad folds (pachygyria). Histopathology demonstrates thickening of the cerebral cortex, altered gray-to-white matter ratio and replacement of a normal 6-layered cortex with a 4-layered disorganized cortex [2, 3]. Two types of lissencephaly can be distinguished in humans: classical lissencephaly (or type I), characterized by thickened brain surface with agyria or pachygyria that results from neuronal migration arrest [4, 5] and cobblestone lissencephaly (or type II), characterized by thin and nodular brain surface, resulting from glial and neuronal overmigration [4, 5]. Muscular dystrophy, ocular alterations, obstructive hydrocephalus, and malformation of the brainstem and cerebellum are often associated with cobblestone lissencephaly [4–6]. Lissencephaly has been described in Lhasa Apso [7, 8], Pekingese [9], Australian Kelpie [10], Wire-haired Fox Terrier [11], Irish Setter [11], and mixed-breed dogs [12]. In humans, lissencephaly is associated with gene mutations related to brain development or cerebral metabolism [1, 5, 13]. In addition, nongenetic causes, such as intrauterine viral infections, vascular events (hypoxia or hypoperfusion), and maternal metabolic disorders that interrupt cortical formation, have been described [2, 3, 13]. The neurological signs in dogs commonly begin with an early-onset of seizures and behavioral alterations, leading to disability [7, 8, 10]. Clinical findings and magnetic resonance imaging (MRI) features of lissencephaly in Shih Tzu dogs have not been reported previously, and reports of concomitant brain malformations are scarce.

## Case presentation

Four apparently unrelated Shih Tzu dogs were presented with lissencephaly between 2011 and 2018 at the Veterinary Neurology Service of São Paulo State University (UNESP), Brazil. Details regarding clinical features, neurolocalization, ancillary diagnostics and antiepileptic treatment are shown in Table 1. MRI was performed using a 0.25 Tesla scanner (Vet-MR Grande, Esaote, Italy) in all dogs to obtain T1-weighted, T2-weighted, fluid-attenuated inversion recovery (FLAIR) and postcontrast T1 sequences. In addition, gradient echo (GRE) sequences were obtained in three dogs, and hybrid contrast enhancement (3D HYCE) sequences were obtained in two dogs. All MRIs were evaluated and interpreted by two researchers (RA and VM). A summary of the MRI findings, equipment, positioning, sequences, imaging parameters, and the contrast medium are detailed in Additional file 1.

**Table 1:** Clinical features, neurolocalization, ancillary diagnostics and antiepileptic therapy in dogs with lissencephaly

	Dog 1	Dog 2	Dog 3	Dog 4
Age (months)	8 m	43 m	18 m	18 m
Sex	Female	Male	Male	Female
Onset of neurological signs	6 m	24 m	12 m	9 m
Neurological presentation	Tonic–clonic seizures and cluster seizures	Tonic–clonic seizures and cluster seizures; difficulties in learning basic commands; behavioral alterations (changes in sleep cycle and aggressiveness) compulsive pacing; bilateral central blindness and bilateral ventromedial strabismus	Tonic–clonic seizures and cluster seizures; behavioral changes (abnormal vocalizations and aggressiveness) and central blindness	Tonic–clonic seizures; cluster seizures, behavioral changes (abnormal vocalizations and aggressiveness during handling); compulsive pacing; bilateral central blindness and bilateral ventromedial strabismus
Neurolocalization	Forebrain lesion	Forebrain lesion	Forebrain lesion	Forebrain lesion
Ancillary diagnostics	Hematological and serum biochemistry profiles were normal. PCR for CDV in urine and IFAT for *T. gondii and N. caninum* in the serum were negative	Hematological and serum biochemistry profiles were normal except for increased level of alkaline phosphatase (216, reference interval 20–156 U/L). PCR for CDV in urine and IFAT for *T. gondii and N. caninum* in the serum were negative	Hematological and serum biochemistry profiles were normal. PCR for CDV in urine and IFAT for *T. gondii and N. caninum* in the serum were negative	Hematological and serum biochemistry profiles were normal. PCR for CDV in urine and IFAT for *T. gondii and N. caninum* in the serum were negative
Antiepileptic therapy	Phenobarbital^a^ 2.5 mg/kg orally q12h prior to referral, increased to 3 mg/kg orally q12h after referral. Serum concentration was not tested due to good control of seizures Levetiracetam^b^ 20 mg/kg, orally q8h for 4 weeks as adjunct therapy for the control of isolate and cluster seizures	Phenobarbital^a^ 2.5 mg/kg orally q12h prior to referral, increased to 4 mg/kg orally q12h after referral KBr 30 mg/kg, orally q24h as adjunct to phenobarbital Levetiracetam^b^ 20 mg/kg, orally q8h for 4–6 weeks as adjunct therapy for the control of isolate and cluster seizures	Phenobarbital^a^ 6 mg/kg orally q12h prior to referral, maintained after referral. Serum concentration was not tested due to financial constraints KBr 40 mg/kg orally q24h prior to referral, reduced to 30 mg/kg after referral as adjunct to phenobarbital	Phenobarbital^a^ 2 mg/kg orally q12h prior to referral, increased to 2.7 mg/kg orally q12h after referral. Serum concentration was not tested due to financial constraints Levetiracetam^b^ 20 mg/kg orally q8h for 4 weeks as adjunct to avoid cluster and isolated seizures on presentation
Follow-up	Alive at 24 months of age Nonprogressive neurological signs Absence of cluster seizures Persistence of isolate epileptic seizures	Alive at 36 months of age Nonprogressive neurological signs Absence of cluster seizures Persistence of isolate epileptic seizures, behavioral changes, central blindness, and strabismus	Alive at 12 months of age Nonprogressive neurological signs Absence of cluster seizures Persistence of isolate epileptic seizures, behavioral changes, and central blindness	Alive at 12 months of age Nonprogressive neurological signs Absence of cluster seizures Persistence of behavioral changes, central blindness, and strabismus

*PCR* polymerase chain reaction, *CDV* canine distemper virus, *IFAT* indirect immunofluorescence antibody test, *KBr* potassium bromide

^a^Gardenal^®^, Safoni, Brazil

^b^Keppra^®^, UCB Biopharma, Brazil

### Case 1

The first case was an 8-month-old spayed female referred in 2011 due to seizures of suspected idiopathic origin that were poorly controlled with phenobarbital (Gardenal^®^, 2.5 mg/kg, orally q12h; Safoni, Brazil). The dog was evaluated by the referring veterinarian 2 months after the onset of tonic–clonic seizures, which had progressed over the last 2 weeks to 2–3 seizures per week. In our service, the owner reported that the dog experienced cluster seizures that occurred over 48 h and were treated with diazepam and thiopental. On presentation, neurological examination was performed 1 week after cluster seizures and was unremarkable. Based on a history of seizures, the lesion was localized to the forebrain. Physical examination findings, biochemical profile, and complete blood count (CBC) were normal; polymerase chain reaction (PCR) for canine distemper virus in urine and indirect immunofluorescent antibody tests (IFAT) for antibodies against *Toxoplasma gondii* and *Neospora caninum* were negative.

**Fig. 1:**
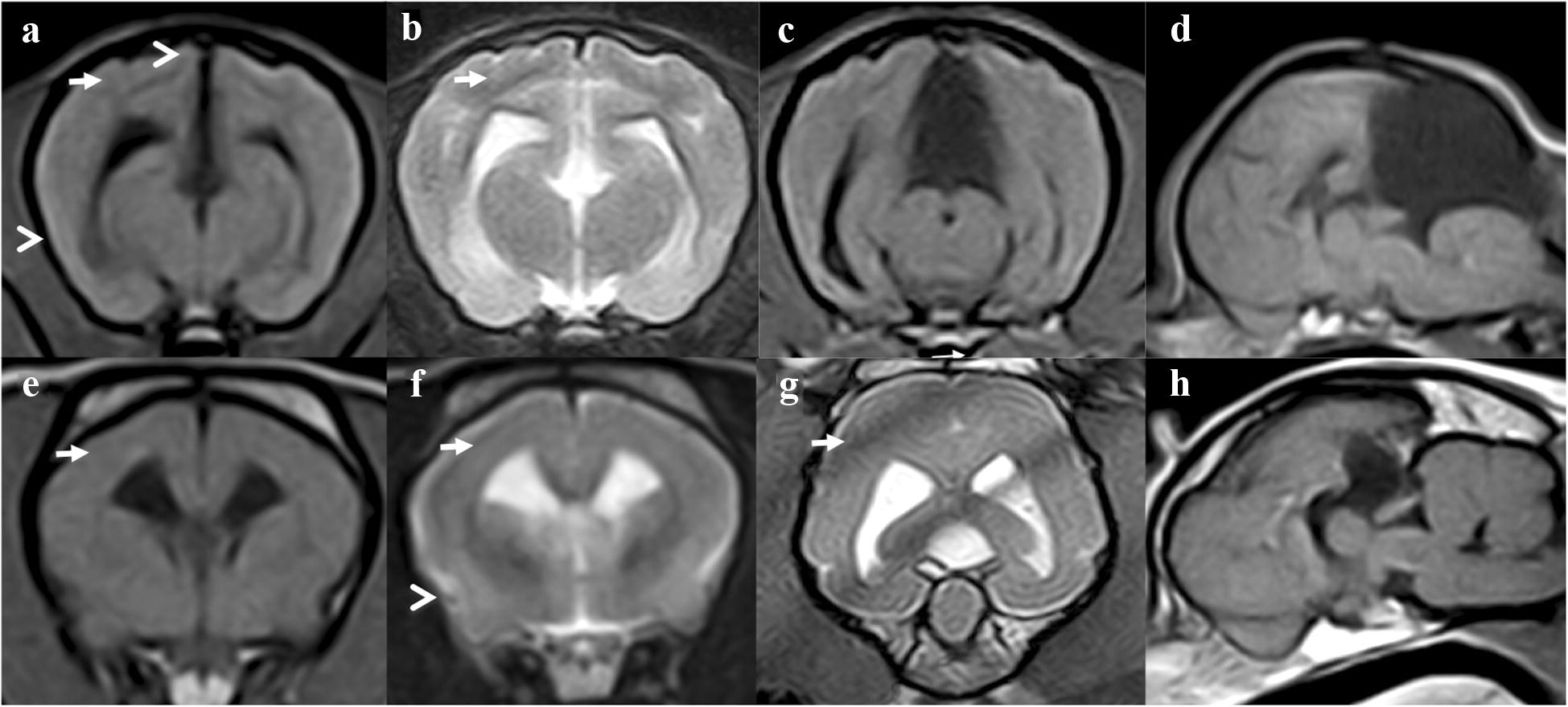
Brain magnetic resonance imaging (MRI) in Shih Tzu dogs with lissencephaly. Transverse T1-weighted (**a**, **e**), T2-weighted (**b**, **f**), fluid-attenuated inversion recovery (FLAIR) (**c**), dorsal hybrid contrast enhancement (3D HYCE) (**g**) and mid-sagittal T1-weighted (**d**, **h**) imaging of the first and second cases. MRI of the first dog showed broad folds with simplified patterns and shallow grooves (pachygyria) in the frontal and parietal lobe region (arrow). Absence of the marginal gyri, middle ectomarginal gyri and caudal suprasylvian gyri was observed in these regions (arrowhead) (**a**, **b)**. Ventriculomegaly and enlargement of the sole quadrigeminal cistern (type SFA-QC) was apparent (**c**, **d**). MRI of the second dog showed diffuse agyria (arrows) at the level of the interthalamic adhesion (**e**, **f**). Rudimentary lateral rhinal sulci were present (arrowhead), and cingulate gyri were not apparent. The internal capsule was abnormally small (**e**, **f)**. Smooth cortical appearance was observed with a lack of the marginal gyri, middle ectomarginal gyri and middle suprasylvian gyri. Dorsocaudal outpocketing of the third ventricle (type SFA-III) and internal hydrocephalus were visualized **(g**, **h**)

MRI showed mixed parieto-occipital agyria and pachygyria of the frontal and parietal lobes (Fig. 1). Temporal and occipital regions lacked gyri and sulci. MRI diagnosis indicated lissencephaly and supracollicular fluid accumulation (SFA) (Fig. 1). Treatment with phenobarbital (Gardenal^®^, 3 mg/kg, orally q12h; Safoni, Brazil) and levetiracetam as an adjunct (Keppra^®^, 20 mg/kg, orally q8h; UCB Biopharma, Brazil) effectively controlled cluster seizures after presentation. Levetiracetam was discontinued after 4 weeks. The neurological signs were nonprogressive and this dog experienced only isolated episodes (interictal period of 2–3 months) over a period of 24 months after diagnosis of lissencephaly (> 50% reduction in the frequency of seizures). It was not possible to obtain information regarding survival for this dog.

### Case 2

The second case involved a 43-month-old castrated male which was referred in 2014 due to the occurrence of cluster seizures. The dog was diagnosed by the referring veterinarian with presumed idiopathic epilepsy at 24 months of age and treatment with phenobarbital was then initiated (Gardenal^®^, 2.5 mg/kg, orally q12h; Safoni, Brazil). During the first 16 months after diagnosis of presumed idiopathic epilepsy and the onset of treatment, the dog experienced both isolated seizures and cluster seizures, with an interictal period less than 30 days. Relatively low levels of phenobarbital (< 25 µg/mL: therapeutic window 25–35 µg/mL) were detected in serum; therefore, the dose of phenobarbital was gradually increased (Gardenal^®^, 4 mg/kg, orally q12h; Safoni, Brazil). However, despite the increase in serum phenobarbital concentration (35 µg/mL), the dog continued to have repeated tonic–clonic seizures. Therefore, potassium bromide (KBr) (30 mg/kg orally, q24h) was also prescribed 1 month before referral to UNESP.

On presentation in our service, in addition to cluster seizures the owner reported behavioral changes between seizures, such as difficulties in learning basic commands, changes in sleep cycle and compulsive pacing. Aggression was noted in our service during manipulation for physical examination. During anamnesis, the owner reported polyuria, polydipsia and polyphagia. Biochemical profile showed increased levels of alkaline phosphatase (216: reference interval 20–156 U/L). These alterations were presumed to be associated with phenobarbital treatment. Neurological examination revealed no abnormalities except for the presence of bilateral central blindness and bilateral ventromedial strabismus during cranial nerve examination. Physical and ophthalmological examinations were normal. The anatomical neurolocalization was compatible with a forebrain lesion. PCR in urine for canine distemper virus and IFAT in the serum for *T. gondii* and *N. caninum* were negative.

MRI showed absence of sulci and gyri with superficial undulations in the frontal and temporal lobes. The main gyri, including the marginal, ectomarginal, suprasylvian, and ectosylvian gyri, were absent. A rudimentary lateral rhinal sulcus was present, while the cingulate gyrus was not apparent. The internal capsule was abnormally small (Fig. 1). A diagnosis of lissencephaly, internal hydrocephalus, corpus callosum hypoplasia, and SFA was established.

This dog remained stable and neurological signs were nonprogressive for 36 months after diagnosis of lissencephaly (interictal interval of 3–4 months with > 50% reduction in the frequency of seizures) using combined polytherapy involving both phenobarbital (Gardenal^®^, 4 mg/kg, orally q12h; Safoni, Brazil) and KBr (30 mg/kg, orally, q24h). Levetiracetam (Keppra^®^, 20 mg/kg, orally q8h for 4–6 weeks, UCB Biopharma, Brazil) was initially included as an adjunct treatment modality. No more cluster seizures were reported after presentation. A carbonic anhydrase inhibitor (acetazolamide, Diamox^®^, 10 mg/kg, orally q8h, Genom, Brazil) and a proton-pump inhibitor (omeprazole, Gaviz^®^, 10 mg/dog, orally q24h, Agener, Brazil) were used for supportive treatment of hydrocephalus. However, the difficulties in learning basic commands, changes in sleep cycle, compulsive pacing, strabismus and aggression were persistent despite treatment. Phone conversation with the owner revealed that the dog was alive 6 years after diagnosis.

### Case 3

The third case involved an 18-month-old intact male which was referred in 2014 due to the occurrence of cluster seizures starting 12 days prior to referral. The dog was previously treated with phenobarbital (Gardenal^®^, 6 mg/kg, orally q12h; Safoni, Brazil); however, due to poor response to treatment, adjunctive therapy with KBr (40 mg/kg, orally, q24h) was initiated. On presentation, anamnesis revealed that 6 months prior to referral, the dog had experienced over 14 isolated seizures within 45 days and subsequent episodes monthly. At evaluation, the dog experienced two tonic–clonic seizures, and emergency treatment was provided using diazepam (1 mg/kg, per rectum and repeated IV bolus 3x). During the 48-h postictal re-evaluation, neurological examination revealed central blindness, and the owner reported that the dog demonstrated abnormal vocalizations. In addition, aggressiveness during the interictal period was noted, mainly during dog handling. Anatomical neurolocalization was consistent with a forebrain lesion. Physical and ophthalmological examination was unremarkable. Laboratory data, including PCR in urine for canine distemper virus and IFAT in the serum for *T. gondii* and *N. caninum* were negative.

**Fig. 2:**
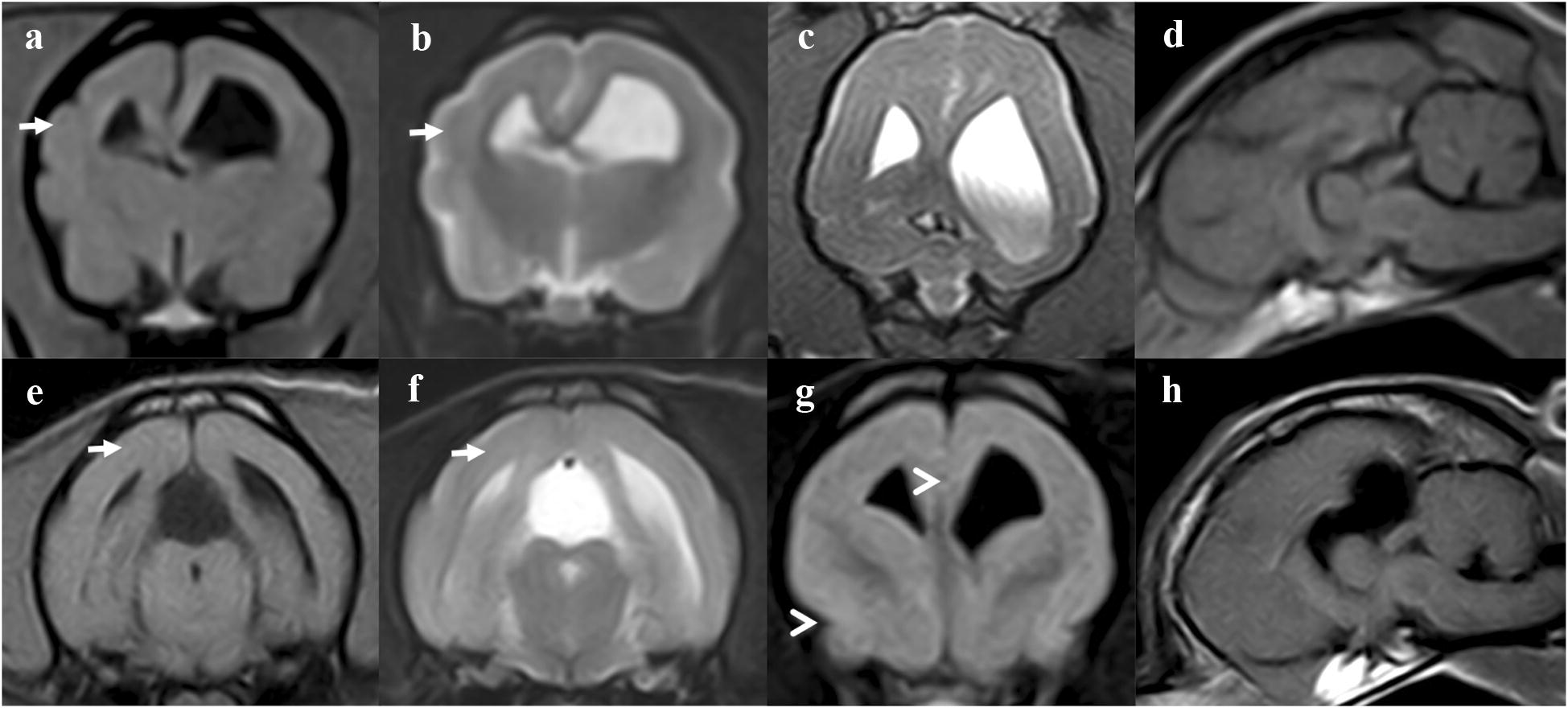
Brain magnetic resonance imaging (MRI) of Shih Tzu dogs with lissencephaly. Transverse T1-weighted (**a**, **e**), T2-weighted (**b**, **f**), dorsal 3D HYCE (**c**), fluid-attenuated inversion recovery (FLAIR) (**g**), and mid-sagittal T1-weighted (**d**, **h**) in the third and fourth cases. MRI of the third dog showed a few sulci in the temporal lobe, including the caudal sylvian gyri and lateral rhinal sulci (arrow). The main gyri (including the marginal, ectomarginal and suprasylvian) and the suprasylvian sulcus were absent. The internal capsule was abnormally small, and internal hydrocephalus was visualized (**a**–**d**). MRI of the fourth dog showed generalized agyria with an absence of sulci and thickened gray matter with smooth appearance (arrow) (**e**, **f**). Rhinal sulci in the temporal lobe were not apparent and cingulate gyrus was absent (arrowhead). Internal hydrocephalus and supracollicular fluid accumulation associated with dorsocaudal outpocketing of the third ventricle (type SFA-III) were observed (**g**, **h**)

MRI of the third dog showed presence of some sulci in the temporal lobe, including the caudal sylvian gyri and lateral rhinal sulci. However, the main gyri, including the marginal, ectomarginal, suprasylvian gyri, and suprasylvian sulcus (division between the parietal and temporal lobe), were absent (Fig. 2). The internal capsule was abnormally small. Diagnosis was consistent with lissencephaly, asymmetrical internal hydrocephalus, and corpus callosum hypoplasia. This dog showed progressive reduction in isolated seizures throughout the year following diagnosis (> 50% reduction in seizure frequency) with an interictal interval of 2 months, maintaining combined polytherapy involving phenobarbital (Gardenal^®^, 6 mg/kg, orally q12h; Safoni, Brazil) and KBr (30 mg/kg, orally, q24h). Serum concentration of phenobarbital was not tested due to financial constraints. No more cluster seizures were observed with combined polytherapy. A carbonic anhydrase inhibitor (acetazolamide, Diamox^®^, 10 mg/kg, orally q8h, Genom, Brazil) and a proton-pump inhibitor (omeprazole, Gaviz^®^, 10 mg/dog, orally q24h, Agener, Brazil) were used for supportive treatment of hydrocephalus. Behavioral changes and central blindness persisted despite treatment. Overall survival was 6 years after diagnosis, confirmed by the owners after our phone call.

### Case 4

The fourth case was an 18-month-old spayed female which was referred with seizures in 2018. The referring veterinarian suspected meningoencephalitis of unknown etiology and reported poor control of seizures with phenobarbital (Gardenal^®^, 2 mg/kg, orally q12h; Safoni, Brazil). Prednisolone treatment (Predsim^®^, 0.5 mg/kg, orally q12h; Medley, Brazil) was added empirically 1 week prior to referral by the referring veterinarian. In our service, the owner reported the first isolated tonic–clonic seizure when the dog was 9 months old. After the interictal period of 9 months, the owner reported that these seizures began again 3 weeks prior to examination. Over the 3-week period before examination, during the interictal periods, subtle behavioral changes such as aggressiveness (mainly after handling), compulsive pacing, abnormal vocalizations, and licking at things were observed. The dog presented with cluster seizures in our service and was treated with diazepam. After stabilization, the postictal state in the following 24 h was characterized by ataxia, paresis, and behavioral signs. Upon neurological examination after postictal stage, consciousness, posture, gait, and postural reactions were normal. Cranial nerve examination showed bilateral central blindness and bilateral ventromedial strabismus. The findings were consistent with a forebrain lesion. Physical and ophthalmological examination findings, biochemical serum profile and CBC test results were unremarkable. Thorax radiography and abdominal ultrasound did not show alterations. PCR in urine for canine distemper virus and IFAT in the serum for *T. gondii and N. caninum* were negative.

MRI showed generalized absence of sulci and gyri and thickened gray matter. The main sulci and gyri, including the marginal, ectomarginal, suprasylvian, ectosylvian, and lateral rhinal sulci, were absent. The cingulate gyrus was not recognizable, and the subcortical internal capsule was abnormally small (Fig. 2). The diagnosis was consistent with lissencephaly with SFA, internal hydrocephalus and corpus callosum hypoplasia. Prednisolone was then discontinued due to lack of indication. Clinical presentation and MRI did not support a diagnosis of meningoencephalitis. After dose readjustment with phenobarbital (Gardenal^®^, 2.7 mg/kg, orally q12h; Safoni, Brazil) and levetiracetam as an adjunct (Keppra^®^, 20 mg/kg, orally q8h for 4 weeks; UCB Biopharma, Brazil), seizure frequency was reduced for the first 3 months (> 50% frequency reduction of seizures). Adjunct treatment with levetiracetam was discontinued after approximately 4 weeks. At the 12-month follow-up examination, seizure reduction with an interictal interval over 2–3 months was noted and no more cluster seizures were observed. However, the behavioral changes, blindness and strabismus observed were persistent despite treatment. A phone conversation with the owners revealed that the dog was alive at the time of writing this report 2 years after diagnosis.

## Discussion and conclusions

Malformations of cortical development have rarely been reported in dogs, but epileptic seizures seem to be a frequent clinical sign in affected dogs [7, 9, 10]. Lissencephaly represents an uncommon disorder of the cortical gyration in dogs [7, 9, 10]. Lissencephaly without concurrent intracranial malformations was described in Lhasa Apso dogs, in an Australian Kelpie dog and in a small mixed-breed dog [7, 10, 12]. This report is the first on lissencephaly in Shih Tzu dogs with other concurrent intracranial malformations and the first report providing a detailed neuroanatomical description of lissencephaly identified on MRI.

In humans, malformations of cortical development can lead to lissencephaly type I, characterized by pachygyria or agyria with thickened and smooth brain surface [4, 5]. Apart from lissencephaly, cortical development disorders that may cause epilepsy as subcortical band heterotopia, polymicrogyria, and cobblestone malformations have been described [1, 2]. Such disorders are rarely reported in dogs [14].

Four Shih Tzu dogs without any known close relationship, were referred because of epileptic seizures. The dogs were not selected among breeds. The onset of seizures was at the age of 6, 9, 12 and 24 months. Previous studies have reported the onset of seizures in dogs between the age of approximately 12 months and 5 years in two Lhasa Apso [7, 8], one Pekingese [9], one Australian Kelpie [10], and one mixed-breed dog [12]. In humans with lissencephaly, approximately 83% of patients experience early‐onset seizures earlier than 7-months-old [15].

Neuro-ophthalmological abnormalities have been poorly characterized in dogs with lissencephaly. Central blindness was previously reported [7, 9]. We detected central blindness in three out of four dogs. Bilateral vision deficits were thought to derive from occipital lobe lesions. Bilateral ventromedial strabismus (esotropia) was detected in two out of four dogs. Only one other report has described ventromedial strabismus in a dog with lissencephaly, which was associated with an orbital anatomical abnormality or short medial rectum muscle [7]. We assumed that maldevelopment of the primary visual cortex and visual motor control mechanism may lead to esotropia [16]. Humans with lissencephaly have visual abnormalities, including no ocular fixation or tracking, poor visual tracking, nystagmus, variable esotropia, oculomotor apraxia, optic nerve and macular hypoplasia/atrophy, delayed visual maturation, and cortical visual impairment [17]. Approximately 67% of human patients with lissencephaly type I have neuro-ophthalmological abnormalities [17].

Severe mental retardation may be observed in humans with lissencephaly and is associated with neuronal migration defects in spatial learning areas [3, 5, 18]. Motor disability as early hypotonia, spastic tetraplegia and opisthotonos is observed in human cases of lissencephaly due to defects in motor areas [3, 5]. Humans with lissencephaly often die before adulthood [1, 6, 17]. In our study, early onset of several behavioral changes was detected in three dogs between 6 and 24 months old. Alterations in locomotor behavior (pacing and changes in sleep pattern), aggressiveness (irritability to manipulation) and vocalization in addition to seizures were observed. Previous studies observed late-onset behavioral changes in dogs with lissencephaly over 12-months-old [7–10, 12], aggressiveness being frequently reported [7, 9, 10]. The different clinical pictures between human and canine lissencephaly could be explained by the fact that the cerebral cortex is less essential in dogs than in humans for motor function [11]. In dogs, motor function may be maintained despite frontoparietal lobe (motor area) and pyramidal system lesions. However, cognitive and learning abilities may be affected [11].

Magnetic resonance imaging in dogs with lissencephaly showed thickened cortical gray matter with smooth appearance, an abnormally small internal capsule, and absence of the major gyri and sulci when compared with healthy Shih Tzu dogs (Additional file 2). Previous studies did not report detailed neuroanatomical examinations of cortical gyri and sulci in dogs with lissencephaly [7, 9, 10]. In addition, MRI was informative for correct *ante*-*mortem* diagnosis of multiple congenital anomalies, determination of cerebral morphology, and the degree of lissencephaly in Shih Tzu dogs.

Lissencephaly is graded in humans using a 6-point grading system based on the severity and anterior–posterior brain gradient of the abnormalities. Grade 1 represents severe lissencephaly with complete agyria, while grade 6 represents mild subcortical band heterotopia [3, 5]. Grades 1a to 6a are more severe posteriorly and 1b to 6b anteriorly [3, 5]. Using these criteria, the second (Fig. 1e), third (Fig. 2a) and fourth (Fig. 2e) cases presented here were classified as grade 2a with diffuse agyria and few shallow undulations in the frontal and temporal lobes. The first dog was classified as grade 3a due to mixed parieto‐occipital agyria and frontal pachygyria (Fig. 1a). Previous studies have reported lissencephaly grade 2a [9] and 2b [10] in dogs. In our study, behavioral alterations and central blindness were noted in dogs with more severe lissencephaly grade, indicating a possible correlation between MRI severity and clinical signs. The grade of lissencephaly was not related to the severity of the clinical signs in previous studies [9, 10].

Two forms of lissencephaly have been described in humans: classic lissencephaly (or type I), characterized by abnormal thick cortical layers (four layered, with a cell-sparse zone), agyria or pachygyria without malformation of the brainstem and cerebellum [2]. Mutations in cytoskeletal genes, such as the platelet activating factor acetylhydrolase 1b regulatory subunit 1, doublecortin, and tubulin A1A genes [1, 2, 5], can lead to lissencephaly in humans and mice [1, 5]. Cobblestone lissencephaly (type II) is characterized by multiple shallow furrows, a thin cerebral mantle and malformation of the brainstem and the cerebellum [2, 5]. In dogs, genetic mutations for lissencephaly have not yet been described [7, 11]. In our study, muscular and ocular disorders were not observed and MRI did not showed nodular brain surface and brainstem or cerebellum malformations associated to cobblestone lissencephaly [4–6]. Therefore, our dogs likely have lissencephaly analogous to type I human lissencephaly [2, 5, 19]. Lissencephaly is overrepresented in the Lhasa Apso dogs and was also reported in the genetically related Pekingese breed [7, 9]. Considering the genetic relation among Lhasa Apso, Pekingese and Shih Tzu breeds [20], it is possible that lissencephaly may be a genetic disease in dogs.

Hydrocephalus and SFA are intracranial malformations most often reported in young and toy breed dogs [21–23]. Enlarged ventricles (a condition known as ventriculomegaly) comprise a common finding in adult brachycephalic dogs [21, 23]. Dogs with only ventriculomegaly do not have increased intraventricular pressure and are considered to be asymptomatic [21, 23] In our study, internal hydrocephalus was confirmed based on specific MRI features that indicated increased intraventricular pressure in three dogs [21]. Hydrocephalus with concomitant lissencephaly has not been reported in dogs. In dogs, lissencephaly has been diagnosed together with ventriculomegaly rather that with hydrocephalus [7, 10, 12]. In a human study, ventriculomegaly has been observed in 73.7% of patients with lissencephaly type I [15]. Supracollicular fluid accumulation without concomitant lissencephaly has been reported in male brachycephalic dogs, and the Shih Tzu dog breed was most often reported [22]. In dogs, most SFAs are associated with dorsocaudal outpocketing of the third ventricle (SFA-III) [24]. An expansion of both the third ventricle and the quadrigeminal cistern is another type of SFA (SFA III-QC) [24]. A few cases present with enlargement of the sole quadrigeminal cistern (SFA-QC) [24]. Previously, it was hypothesized that type SFA-III in predisposed breeds can be part of hydrocephalus rather than an anomaly itself [24]. In our study, the first dog presented with SFA-QC and type SFA-III was detected in the second and fourth dogs. Although SFA can be related to the presence of neurological signs in dogs with lissencephaly, the clinical significance of SFA is variable and SFA may be incidental in dogs with other intracranial diseases [22]. There is no reported genetic relationship between SFA and lissencephaly in neither dogs nor humans. In humans, supracollicular fluid accumulation is associated with a defect in leptomeninges development [19].

Corpus callosum abnormalities have been sporadically reported and are still poorly understood in dogs, typically being an isolated abnormality or associated with holoprosencephaly and inborn errors of metabolism. The most frequent clinical signs described are hypodipsia/adipsia, tremors, and seizures [25]. We observed concurrent corpus callosum hypoplasia in the second, third, and fourth dogs, which is not commonly reported in dogs with lissencephaly [11]. Epileptic seizures may be related to corpus callosum hypoplasia. In humans, corpus callosum abnormalities are associated with classic lissencephaly (type I), cobblestone lissencephaly (type II) and polymicrogyria [2, 15].

The antiepileptic drugs used resulted in good control of cluster seizures with mild adverse effects in the long-term follow-up (12–36 months). Seizures were persistent, although, compared to pretreatment, a reduction in the frequency (> 50% or more) and severity of seizures as well as cessation of cluster seizures was observed. Other signs of forebrain abnormalities, such as behavioral alterations, central blindness, and bilateral strabismus were persistent.

Several medications, including carbonic anhydrase inhibitor acetazolamide and proton-pump inhibitor omeprazole, with the goal of decreasing cerebrospinal fluid production, have been proposed as medical management in dogs with hydrocephalus [23, 26, 27]. Experimental studies in healthy dogs and rabbits reported that cerebrospinal fluid production was reduced after acetazolamide and omeprazole treatment, respectively [26, 28]. Due to hydrocephalus, the second and third dogs were medically treated with acetazolamide and omeprazole. Nevertheless, aggressiveness, changes in sleep cycle, compulsive pacing, central blindness, and strabismus were persistent during the follow-up period despite treatment with acetazolamide and omeprazole. One previous report has described no significant effects on recovery of the neurological signs or ventricular volume reduction after treatment with acetazolamide in dogs with hydrocephalus [29]. Furthermore, chronic oral omeprazole therapy in healthy dogs did not affect cerebrospinal fluid production [30].

The major limitation of this study was the lack of histopathological evaluation, which was not possible because dogs were not euthanized during the follow-up period.

Although transcranial ultrasonography or computed tomography may aid in diagnosis, these methods are not precise [9, 10]. In our study, the use of low-field MRI provided a good resolution for *ante*-*mortem* diagnosis of lissencephaly in all dogs. Variations in signal intensity, identification of abnormal cortical layers and depth of cortical sulci were observed. All anatomical structures of the cerebral cortex were identified (Additional file 2). In agreement, previous studies performed diagnosis in dogs using MRI fields between 0.4- and 1.5-Tesla [7, 9, 10]. MRI is the modality of choice for lissencephaly diagnosis and for the differentiation of other neuronal migration disorders, showing a correlation with histopathological features in dogs and humans [1, 2, 7].

Lissencephaly should be considered an important differential diagnosis in Shih Tzu dogs presenting predominantly with early-onset signs of forebrain abnormalities, including tonic–clonic seizures, behavioral alterations, central blindness, and bilateral ventromedial strabismus. Low-field MRI may be a useful diagnostic tool to detect cases of lissencephaly. Hydrocephalus, SFA, and corpus callosum hypoplasia comorbidities could also be associated with lissencephaly in Shih Tzu dogs.

## Supplementary information

**Additional file 1: Table S1.** DOC. Summary of magnetic resonance imaging (MRI) findings of lissencephaly and concomitant congenital malformations in Shih Tzu dogs. Details regarding malformation type, MRI scan, positioning of the patient, sequence types, imaging parameters and contrast medium are described.

**Additional file 2: Figure S1.** DOC. Brain magnetic resonance imaging (MRI) in a healthy Shih Tzu dog. Transverse T1-weighted (**a** and **b**) and transverse and sagittal T2-weighted (**c** and **d**) imaging. The following structures were identified at the level of the interthalamic adhesion: marginal gyri (a); marginal sulci (b); middle ectomarginal gyri (c); ectomarginal sulci (d); middle suprasylvian gyri (e); middle suprasylvian sulci (f); middle ectosylvian gyri (g); caudal ectosylvian sulci (h); caudal sylvian gyri (i); pseudosylvian fissure (j); lateral rhinal sulci (k); splenial sulci (l); cingulate gyri (m) and corpus callosum (o) (**a**). The following structures were identified at the level of the mesencephalic aqueduct: marginal gyri (a); marginal sulci (b); middle ectomarginal gyri (c); ectomarginal sulci (d); caudal suprasylvian gyri (e); caudal suprasylvian sulci (f); ectosylvian gyri (g); lateral rhinal sulci (h); parahippocampal gyri (i) and caudal composite gyri (j) (**b**). In transverse and sagittal T2-weighted images, all anatomical structures were normal, including the lateral ventricles, quadrigeminal cistern and corpus callosum (**c** and **d**).

## Data Availability

The datasets used and/or analyzed during the current study are available from the corresponding author on reasonable request.

## Abbreviations

CBC: Complete blood count
CDV: Canine distemper virus
FLAIR: Fluid-attenuated inversion recovery
GRE: Gradient echo
KBr: Potassium bromide
IFAT: Indirect immunofluorescent antibody test
MRI: Magnetic resonance imaging
PCR: Polymerase chain reaction
SFA: Supracollicular fluid accumulation
3D HYCE: Hybrid contrast enhancement
