# Supplementary material for: Lissencephaly in Shih Tzu dogs
Source: Acta Vet Scand. 2020 Jun 20;62:32. doi: 10.1186/s13028-020-00528-0 (PMC7305484; doi:10.1186/s13028-020-00528-0)
Supplement: Supplementary file 1 — Additional file 1: Table S1. DOC. Summary of magnetic resonance imaging (MRI) findings of lissencephaly and concomitant congenital malformations in Shih Tzu dogs. Details regarding malformation type, MRI scan, positioning of the patient, sequence types, imaging parameters and contrast medium are described. [file 13028_2020_528_MOESM1_ESM.docx]

| **Table S1.** Summary of magnetic resonance imaging (MRI) findings of lissencephaly and concomitant congenital malformations in Shih Tzu dogs. Details regarding malformation type, MRI scan, positioning of the patient, sequence types, imaging parameters and contrast medium are described. | | | | |
| --- | --- | --- | --- | --- |
|  | **Dog 1** | **Dog 2** | **Dog 3** | **Dog 4** |
| MRI equipment, positioning, | O.25T (Vet-MR Grande, Esaote, Italy); sternal recumbency using brain coil 2 | O.25T (Vet-MR Grande, Esaote, Italy); lateral recumbency using brain coil 4 | O.25T (Vet-MR Grande, Esaote, Italy); lateral recumbency using brain coil 4 | O.25T (Vet-MR Grande, Esaote, Italy); lateral recumbency using brain coil 4 |
| sequences, imaging parameters and contrast media | T1-weighted pre-and post-contrast trans (TE 26 ms, TR 850 ms, FOV 256 x 256 mm and slice thickness 4mm); T2-weighted trans (TE 90 ms, TR 5440 ms, FOV 512 x 512 mm and slice thickness 4mm); FLAIR trans (TE 100ms, TR 250ms, FOV 256 x 256 mm and slice thickness 4.5mm); gadoteric acid^a^ 0,1mmol/kg IV | T1-weighted pre-and post-contrast trans (TE 18 ms, TR 500 ms, FOV 256 x 256 mm, and slice thickness 3mm); T2-weighted trans (TE 90 ms, TR 3800 ms, FOV 256 x 256 mm, and slice thickness 3mm); FLAIR trans (TE 90 ms, TR 9320 ms, FOV 256 x 256 mm and slice thickness 3mm); GRE trans (TE 22 ms, TR 1300 ms, FOV 512 x 512 mm and slice thickness 3mm); 3D HYCE high resolution trans, sag and dorsal (TE 5 ms, TR 10 ms, FOV 512 x 512 mm, and slice thickness 0,6-0,8mm); gadoteric acid^a^ 0,1mmol/kg IV | T1-weighted pre-and post-contrast trans (TE 18 ms, TR 500 ms, FOV 256 x 256 mm and slice thickness 3mm); T2-weighted trans (TE 90 ms, TR 4130 ms, FOV 256 x 256 mm, and slice thickness 3mm); FLAIR (TE 90 ms, TR 9030 ms, FOV 512 x 512 mm and slice thickness 3 mm); GRE trans (TE 22 ms, TR 1300 ms, FOV 512 x 512 mm, and slice thickness 3mm); 3D HYCE high resolution trans and sag (TE 5 ms, TR 10 ms, FOV 512 x 512 mm and slice thickness 0,6-0,7mm); gadoteric acid^a^ 0,1mmol/kg IV | T1-weighted pre-and post-contrast trans (TE 18 ms, TR 600 ms, FOV 512 x 512 mm, and slice thickness 4mm); T2-weighted trans (TE 90 ms, TR 3120 ms, FOV 512 x 512 mm and slice thickness 4 mm); FLAIR trans (TE 90 ms, TR 9910 ms, FOV 512 x 512 mm and slice thickness 4mm) and GRE trans (TE 22 ms, TR 1300 ms, FOV 512 x 512 mm and slice thickness 4mm); gadoteric acid^a^ 0,1mmol/kg IV |
| 6-point grading system of lissencephaly | Grade 3a due to mixed parieto‐occipital agyria and pachygyria in the frontal and parietal lobes | Grade 2a with diffuse agyria and few shallow undulations in the frontal and temporal lobes | Grade 2a with diffuse agyria and few shallow undulations in the temporal lobe | Grade 2a with diffuse agyria and few shallow undulations in the temporal lobe |
| Supracollicular fluid accumulation (SFA) | Yes | Yes | No | Yes |
| SFA type | Expansion of third ventricle and quadrigeminal cistern (SFA-QC) | Dorsocaudal outpocketing of the third ventricle (SFA-III) | No | Dorsocaudal outpocketing of the third ventricle (SFA-III) |
| Hydrocephalus | No | Expansion of ventricular system including lateral and third ventricles; flattening of the interthalamic adhesion and diminished suprasellar cistern; presence of dilation of the olfactory recess; increase of ventricle/brain index | Expansion of ventricular system including lateral and third ventricles; flattening of the interthalamic adhesion and diminished suprasellar cistern; mild periventricular white matter edema; increase of ventricle/brain index | Expansion of the ventricular system (including lateral ventricles); width of fastigial recess indicating expansion of the fourth ventricle; flattening of the interthalamic adhesion; diminished suprasellar cistern; mild periventricular white matter edema |
| Corpus callosum hypoplasia | No | Hypoplasia | Hypoplasia | Hypoplasia |

*MRI* magnetic resonance imaging, *FOV* field of view, *TE* echo time, *TR* repetition time, *FLAIR* fluid attenuated inversion recovery*, GRE* gradient echo, *Trans* transverse, *Sag* sagittal, *HYCE* hybrid contrast enhancement, *SFA* supracollicular fluid accumulation, *CSF* cerebrospinal fluid, *QC* quadrigeminal cistern, *III* third ventricle.

^a^ Dotarem^®^, Guerbet Ltd., Brazil.
