# Supplementary material for: Lissencephaly in Shih Tzu dogs
Source: Acta Vet Scand. 2020 Jun 20;62:32. doi: 10.1186/s13028-020-00528-0 (PMC7305484; doi:10.1186/s13028-020-00528-0)
Supplement: Supplementary file 2 — Additional file 2: Figure S1. DOC. Brain magnetic resonance imaging (MRI) in a healthy Shih Tzu dog. Transverse T1-weighted (a and b) and transverse and sagittal T2-weighted (c and d) imaging. The following structures were identified at the level of the interthalamic adhesion: marginal gyri (a); marginal sulci (b); middle ectomarginal gyri (c); ectomarginal sulci (d); middle suprasylvian gyri (e); middle suprasylvian sulci (f); middle ectosylvian gyri (g); caudal ectosylvian sulci (h); caudal sylvian gyri (i); pseudosylvian fissure (j); lateral rhinal sulci (k); splenial sulci (l); cingulate gyri (m) and corpus callosum (o) (a). The following structures were identified at the level of the mesencephalic aqueduct: marginal gyri (a); marginal sulci (b); middle ectomarginal gyri (c); ectomarginal sulci (d); caudal suprasylvian gyri (e); caudal suprasylvian sulci (f); ectosylvian gyri (g); lateral rhinal sulci (h); parahippocampal gyri (i) and caudal composite gyri (j) (b). In transverse and sagittal T2-weighted images, all anatomical structures were normal, including the lateral ventricles, quadrigeminal cistern and corpus callosum (c and d). [file 13028_2020_528_MOESM2_ESM.docx]

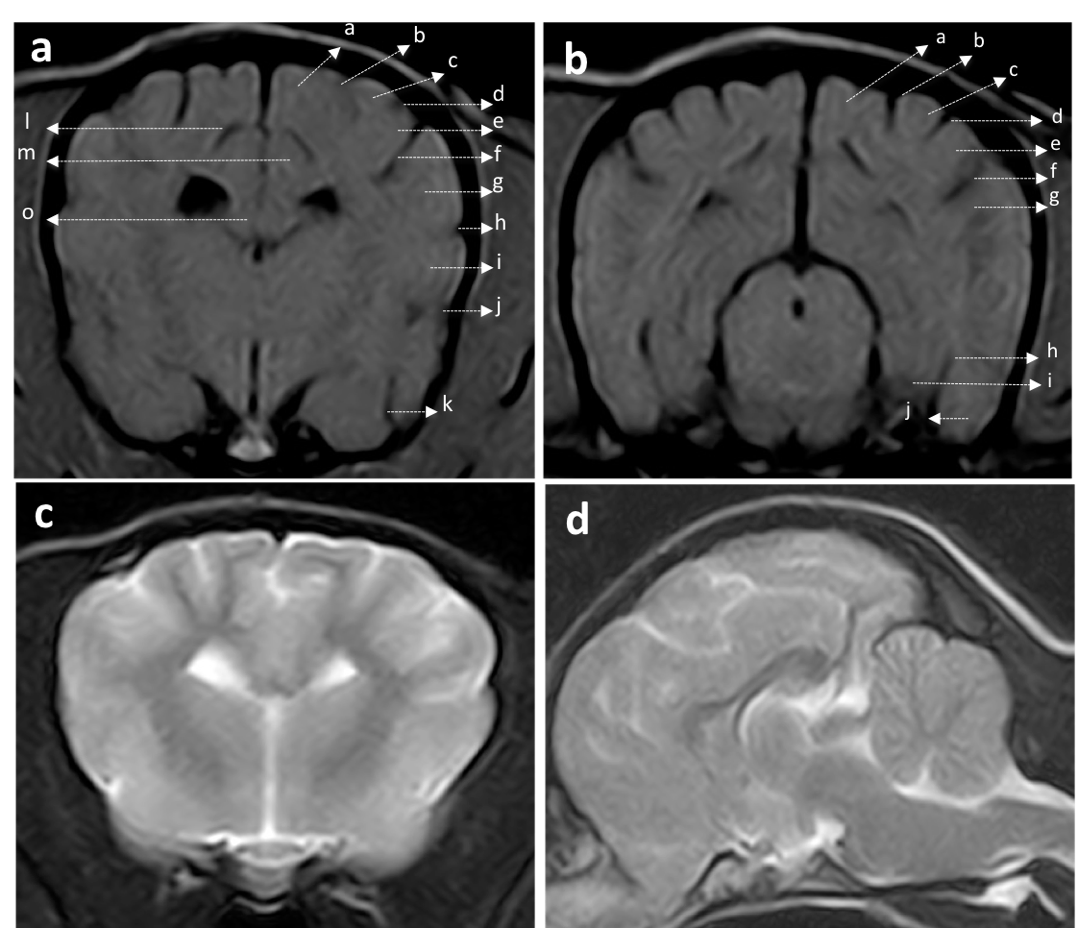


**Figure S1.** Brain magnetic resonance imaging (MRI) in a healthy Shih Tzu dog. Transverse T1-weighted (**a** and **b**) and transverse and sagittal T2-weighted (**c** and **d**) imaging. The following structures were identified at the level of the interthalamic adhesion: marginal gyri (a); marginal sulci (b); middle ectomarginal gyri (c); ectomarginal sulci (d); middle suprasylvian gyri (e); middle suprasylvian sulci (f); middle ectosylvian gyri (g); caudal ectosylvian sulci (h); caudal sylvian gyri (i); pseudosylvian fissure (j); lateral rhinal sulci (k); splenial sulci (l); cingulate gyri (m) and corpus callosum (o) (**a**). The following structures were identified at the level of the mesencephalic aqueduct: marginal gyri (a); marginal sulci (b); middle ectomarginal gyri (c); ectomarginal sulci (d); caudal suprasylvian gyri (e); caudal suprasylvian sulci (f); ectosylvian gyri (g); lateral rhinal sulci (h); parahippocampal gyri (i) and caudal composite gyri (j) (**b**). In transverse and sagittal T2-weighted images, all anatomical structures were normal, including the lateral ventricles, quadrigeminal cistern and corpus callosum (**c** and **d**).
